# The World's Rediscovered Species: Back from the Brink?

**DOI:** 10.1371/journal.pone.0022531

**Published:** 2011-07-27

**Authors:** Brett R. Scheffers, Ding Li Yong, J. Berton C. Harris, Xingli Giam, Navjot S. Sodhi

**Affiliations:** 1 Department of Biological Sciences, National University of Singapore, Singapore, Singapore; 2 Environment Institute and School of Earth and Environmental Sciences, University of Adelaide, Adelaide, Australia; 3 Department of Ecology and Evolutionary Biology, Princeton University, Princeton, New Jersey, United States of America; Smithsonian's National Zoological Park, United States of America

## Abstract

Each year, numerous species thought to have disappeared are rediscovered. Yet, do these rediscoveries represent the return of viable populations or the delayed extinction of doomed species? We document the number, distribution and conservation status of rediscovered amphibian, bird, and mammal species globally. Over the past 122 years, at least 351 species have been rediscovered, most occurring in the tropics. These species, on average, were missing for 61 years before being rediscovered (range of 3–331 years). The number of rediscoveries per year increased over time and the majority of these rediscoveries represent first documentations since their original description. Most rediscovered species have restricted ranges and small populations, and 92% of amphibians, 86% of birds, and 86% of mammals are highly threatened, independent of how long they were missing or when they were rediscovered. Under the current trends of widespread habitat loss, particularly in the tropics, most rediscovered species remain on the brink of extinction.

## Introduction

The world is in the midst of a mass extinction event predominantly caused by human actions such as over-harvesting, habitat loss, and wildlife trade [1]–[2]. Currently, 30% of amphibians, 12% of birds, and 21% of mammals are either threatened or already extinct [3]. Recent analyses suggest that the current extinction rate may be 1,000 times higher than that indicated by background extinction rates, and projected future extinction rates may be ten times greater still [4]. Thus, as humanity continues to deplete the earth's biological wealth, we must consider what we have [5], what we have lost [6], and what we thought we had lost [7]–[9].

Not all species believed to be extinct are extinct. In the wake of rampant habitat loss and degradation [10], biological surveys are critically important for conservation [11] and are commonly deployed in an attempt to document residual biological diversity (e.g., Conservation International's Rapid Assessment Program). In some cases, these surveys are designed to rediscover species that have not been seen for long periods of time or species presumed to be extinct. For example, in 2009, BirdLife International set out to relocate 47 species of birds that have not been seen for up to 184 years [12] and Conservation International recently announced an initiative to relocate 100 lost amphibian species [13]. These initiatives often document new species, and in many cases, species thought to have disappeared are rediscovered.

Extinction is a focal issue among scientists, policy makers, and the general public. Therefore, species rediscoveries are often celebrated by the media and have the potential to generate support for conservation. Rediscoveries can also be controversial and may spur unsupported optimism for the survival of the species. For example, the ivory-billed woodpecker, *Campephilus principalis* was possibly rediscovered in 2005 and the greater akialoa, *Hemignathus ellisianus* was rediscovered in 1960; there have been no confirmed sightings since their rediscovery. In some cases rediscoveries can even lead the general public to believe that the extinction crisis is not as bad as stated or lead to the loss of credibility with the public [14]. Despite the prevalence of rediscoveries in the scientific literature and media, the magnitude of rediscoveries has rarely been quantified (but see [9]).

In the current study we: 1) documented the number of rediscovered amphibian, bird, and mammal species with respect to year rediscovered and geographic location; 2) assessed the current conservation status of these rediscovered species relative to all other species in their taxonomic group and its association with the period of time missing (year rediscovered minus year last seen); and 3) considered two macroecological characteristics, range size and minimum elevation of occurrence, that may have influenced when a species is rediscovered. A species' geographic range may influence the probability of it being rediscovered—species with large ranges are likely to be rediscovered sooner than those with small restricted ranges. Similarly, elevation of occurrence may also influence whether or not a species is rediscovered. For example, two recent studies found that range size and elevation of occurrence were important predictors of whether or not mammal species were rediscovered [9], [15]. Therefore, we predicted that the number of years a species goes missing will be correlated with its range size and minimum elevation of occurrence. We predicted that those with smaller ranges would be missing for longer periods of time than those with large ranges and those that occur at higher elevations would be missing for shorter periods of time than those that occur at higher elevations (*sensu* [9]). Lastly, we determined if the number of years a species goes missing and the year of rediscovery were correlated with whether or not a species was threatened. This relationship may be expected as species that are missing for short periods of time may be less threatened than those species that are missing for longer time periods.

## Materials and Methods

We recorded all rediscovered amphibian, bird and mammal species from peer-reviewed literature and web searches. In total, we reviewed 4991 sources, comprising 2928 peer-reviewed articles and 2063 websites. We used the following search term, (rediscover* and species* and [taxonomic group (i.e., amphibian or bird or mammal)], in ISI Web of Science, BIOSIS, and Zoological Records as well as “rediscovered” and [taxonomic group] in Google Scholar and Google. We included an online Web search (Google Scholar and Google) as many rediscoveries are not published in peer-reviewed journals but are instead reported in grey-literature and/or online. We recorded all species that were considered “rediscovered” by the authors of the reference. All rediscoveries represent global rediscoveries rather than regional rediscoveries of species populations. We validated global rediscoveries based on the author's statement that the species was thought to have been globally missing. If the authors did not explicitly state this we searched other published, independent, sources to validate that the species had disappeared globally. We may not have recorded all rediscoveries and thus, we do not claim that this study is comprehensive but instead indicative. Sources were searched until rediscoveries leveled off. Therefore, we are confident that our documented rediscoveries represent the magnitude of reported rediscoveries accurately due to sampling saturation. Our mammal data were collected independently but are complimentary to Fisher and Blomberg [9]. Fisher and Blomberg [9] quantify mammal rediscoveries; however, they only included published accounts of mammals rediscovered and thus missed many rediscoveries reported in grey literature and/or online. We chose not to include a minimum time period for which a species must be missing in order to be considered rediscovered, but instead relied on expert opinion (the author's or scientist's judgment) to declare a species rediscovered. This is important as there are no accepted guidelines for defining a species rediscovery. Therefore, a species may go undetected for several years without being seen, but if scientists do not declare the species to be lost and subsequently found, the species is not considered rediscovered. Additionally, it might be expected for a very rare species to go undetected for several years whereas the disappearance of an abundant species for a year or two may be alarming. With the exception of two species (*Nipponia nippon* and *Pterodroma madeira*), all species in our database were missing for five or more years.

We define three types of rediscoveries: those that were declared extinct (informally declared extinct by the source of the rediscovery) but rediscovered, those that have gone unseen for extended periods of time (i.e., a species goes unseen with no confirmed sightings for an unusual amount of time), and those that represent first sightings since the type series was collected. All types of rediscoveries were informally declared and/or quantified by the source of the rediscovery. Rediscoveries of type specimens from museum collections and genetic rediscoveries (sometimes called re-descriptions) were not included. Each species found on the internet or without published evidence, was verified by a second independent source (e.g., [3], [16]). We excluded all species that were rediscovered from fossils; therefore we only considered rediscoveries since Linnaeus. The year last seen and year of rediscovery were recorded for each species. If the exact date of rediscovery was not provided via the source, we used the publication date as a relative date of rediscovery. For species that were recorded as last seen during a decade, we chose the middle year of the decade as an approximation; if a species was last seen in the 1980's, we chose 1985 as a representative of the year last seen.

We plotted the total number of species rediscovered per year and per decade. In order to adequately interpret these trends, one must consider the amount of search effort that coincides with rediscoveries. We scaled the raw numbers of species rediscovered, by the “effort” required to relocate them (the number of taxonomists working to describe new species in that particular taxonomic group; data obtained from [3]) to produce the number of species rediscovered per unit time per taxonomists [15]. Here we define “taxonomists” simply as those who describe new species. For each taxonomic group, the number of taxonomists was recorded as the number of first authors in each year. In other words, we recorded the number of first author amphibian taxonomists, bird taxonomists, and mammal taxonomists in each year (starting at the year of the first rediscovery; 1889). We only used first authors in order to avoid inflated author counts due to large numbers of authors that do not typically conduct field research (e.g., molecular phylogeneticists). Generally, the number of new species discovered has increased accordingly with the increase in the number of taxonomists since Linnaeus in the mid-1700's [17]. Therefore, the more taxonomists active in describing new species—the more species we should expect to be rediscovered. “Taxonomist” is defined simply as those who describe species. In this study, we assume that the documentation of rare species will also increase with search effort. However, we recognize that finding rare species is also related to current threat status. Lastly, in order to determine if the rate of rediscovery is similar between threatened and non-threatened species, we plotted accumulation curves over time for threatened, non-threatened, Data Deficient and total species for each taxonomic class through time and visually examined trends.

We examined spatial trends in rediscoveries by overlaying ranges of all rediscovered amphibian and mammal species onto the WWF Ecoregions of the World dataset using ArcGIS 9.3 software. Because range sizes for rediscovered species are small we plotted the distribution of species based on ecoregions in order to examine hotspots of rediscoveries that relate to biologically relevant regions. We used range maps from the Global Amphibian and Mammal assessments [3] to plot the distribution of rediscovered species globally. We acquired range maps for all threatened birds from Birdlife International (www.birdlife.org), which comprises 86% of all birds from this study. Antarctica was excluded as it does not contain any amphibians, mammals, or rediscovered birds, and Alaska (United States) was excluded for display purposes as it does not contain any rediscovered amphibians, mammals, or birds.

We defined the threat status for each rediscovered species, grouped by taxonomic class, according to Schipper et al. [18] and therefore refer to this index as “Schipper's threat level”. We used IUCN 2010 threat categories: EX, Extinct; EW, Extinct in the Wild; CR, Critically Endangered; EN, Endangered; VU, Vulnerable; NT, Near threatened; LC, Least Concern; DD, Data Deficient. We define Schipper's threat level as [(VU+EN+CR)/(Total−DD)]×100, which represents a best estimate of extinction risk (see [18]). The range in Schipper's threat level was calculated as between [(VU+EN+CR)/Total]×100 and [(VU+EN+CR+DD)/Total]×100.

The total number of years a species goes missing may be an indicator of threat status. We determined time missing by subtracting the year last seen from the year of rediscovery. Similarly, the year in which a species is rediscovered may influence the current threat status of a species, as species rediscovered earlier may have had more time to recover. Alternatively, the populations of earlier rediscovered species may have had more time to decline from disturbances. To examine the relationship between 2010 IUCN threat status and the year of rediscovery and number of years gone missing for each species, we took the following two approaches: 1) we summarized the threat status for each taxonomic group in relation to the number of years a species went missing. This allowed us to assess whether or not highly threatened species are missing for shorter or longer periods of time than less threatened species; and 2) we fitted binomial generalized linear models to the data in R 2.11.1 (R Project for Statistical Computing, http://www.r-project.org) to test if the year of rediscovery or the number of years missing predicts whether or not a species is threatened. Our binomial response was coded as threatened or non-threatened. Our response followed IUCN's definition of threat status where Critically Endangered, Endangered, Vulnerable species were classified as threatened and near threatened and Least Concern species were classified as non-threatened. Species classified by IUCN as Data-Deficient were excluded as their conservation status could not be determined due to a lack of data. We used year of rediscovery and number of years missing as predictor variables in our analysis. In order to decipher whether or not trends are similar among taxonomic groups we performed our analyses for all species combined and ran separate models for each taxonomic class. Due to limited time series data on threat status it is possible that some of the species may have previously been more threatened at the time of rediscovery and have since improved in status; however this is likely irrelevant as the majority of species are threatened.

It is possible that species with large ranges went missing for shorter periods of time than those with smaller ranges. To test this, we used Gaussian generalized linear models to assess whether range size was negatively correlated with the number of years a species went missing. Range size was coded as the response variable and years gone missing and taxonomic class as the predictor variables. Taxonomic class was coded as a predictor variable in order to account for potential influence on threat status by a single taxonomic group. We also descriptively summarized the minimum elevation of occurrence and range size for each species. Range size and minimum elevation of occurrence data were collected from the following sources: amphibians [3], [19], birds (www.birdlife.org), and mammals [3], [16], [20].

All models were evaluated in an information-theoretic framework—using Akaike's Information Criterion adjusted for small samples (AIC*_c_*)—to assess the relative strengths of competing candidate models [21]. Relative likelihoods of candidate models were calculated using AIC*_c_* weights, with weights varying from 0 (no support) to 1 (complete support) relative to the entire model set. The amount of variance in the response variable captured by each combination of variables considered was assessed as the percent deviance explained (% DE) [21].

## Results

**Figure 1:**
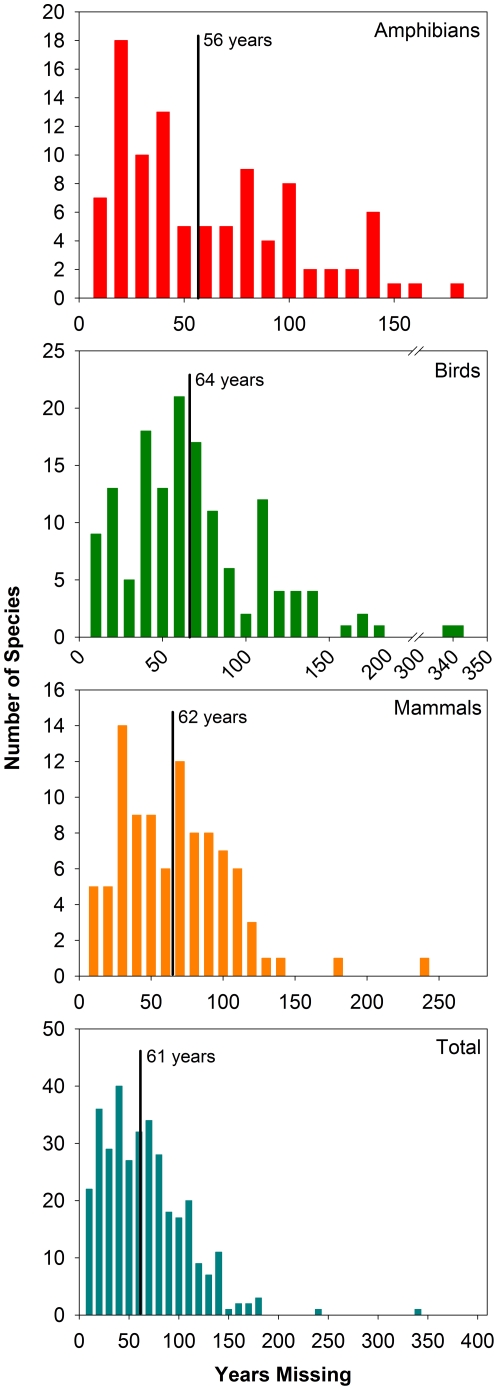
Number of years each species went missing before being rediscovered. The number of years each species went missing before being rediscovered plotted for all amphibian, bird, and mammal species as well as total species (all species combined). Years missing = year rediscovered−year last seen. Black vertical bars indicate mean years missing. Years missing are binned by 10 year increments.

**Figure 2:**
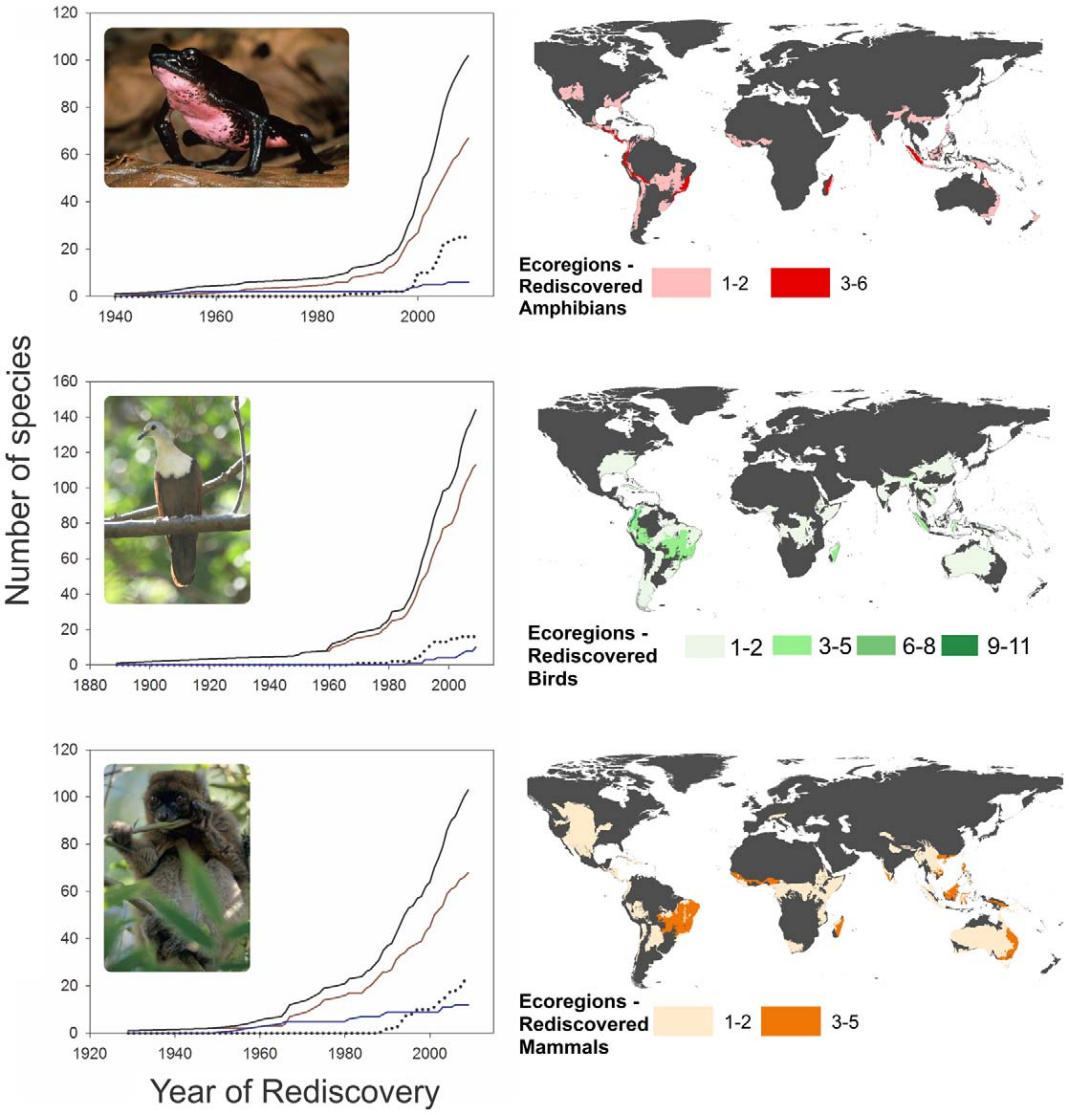
Accumulation threat curves (left) and distribution of rediscovered amphibians, birds and mammals (right). (*Left*) The accumulation of threatened, non-threatened, Data Deficient, and total rediscovered species over the past 122 years. (*Right*) The distribution of rediscovered amphibian, bird, and mammal species globally. Qualitative trends for amphibians, birds, and mammals are presented as those ecoregions that overlap with species ranges. (*Left Figures*) Threatened (red line), includes “Critically Endangered”, “Endangered” and “Vulnerable” species; Non-threatened (blue line) includes “Near Threatened” and “Least Concern” species; Data Deficient (black dotted line) includes “Data Deficient” species, and Total (black solid line) includes Threatened, Non-threatened, and Data Deficient species. Additionally, 3 “Extinct” and 1 “Extinct in the Wild” amphibian species are included in threatened accumulation curves as individuals of each species were recently rediscovered in the wild. *Top photograph*: the Critically Endangered, *Atelopus seminiferus*, rediscovered in Peru in 2001; *middle photograph*: the Endangered, *Gallicolumba hoedtii*, rediscovered in Indonesia in 2008; and *bottom photograph*: the Critically Endangered, *Prolemur simus*, rediscovered in Madagascar in 1986. Photo credits: *A. seminiferus* courtesy of Jan Post, *G. hoedtii* courtesy of Philippe Verbelen. and *P. simus* courtesy of N. Rowe/alltheworldsprimates.org.

We recorded 351 species rediscoveries (Table S1, 104 amphibians, 144 birds, and 103 mammals) over the past 122 years (*Ploceus megarhynchus* (yellow weaver) was the first documented rediscovery, reported in 1889), amounting to approximately 3 species per year. Species went unseen on average for 61 years before being rediscovered (range of 3–331 years) (Figure 1). The majority of amphibians and birds were only known from type specimens prior to their rediscovery (59% and 40% respectively), whereas mammals were predominately thought to have been rediscovered from extinction (Table 1). The number of species rediscovered increased markedly with time for all three groups (Figure S1). The number of rediscoveries per taxonomist increased with time for birds and amphibians, whereas mammal rediscoveries were variable over time (Figure S2). When grouped by decade, however, the number of species rediscovered per taxonomist for all three classes increased with time (Figure S2). Additionally, rediscoveries of threatened species increased exponentially with time, whereas the rediscovery of non-threatened species seemed to saturate (Figure 2). Rediscoveries are concentrated in the lower latitudes and Southern Hemisphere, particularly in tropical and subtropical broadleaf forests of South America (e.g., eastern Cordillera Real forests on the eastern slope of the Andes), Africa (e.g., western Guinean lowland forests), Madagascar (e.g., tropical moist forests), India (e.g., south western Ghats montane rain forests) and New Guinea (central range montane rainforests) (Figure 2). The majority of amphibians were rediscovered at high elevations (>1000 m) whereas birds and mammals were predominately found in the lowlands (<500 m) and foothills (500–1000 m) (Figure S3).

**Table 1:** The number of rediscoveries under three criteria: those that represent first sightings since the type specimen was collected, those that were declared extinct but rediscovered, and those that have gone unseen for extended periods of time.

	Amphibians		Birds		Mammals		Total	
Type	Count	%	Count	%	Count	%	Count	%
Type specimen	61	58.6	58	40.3	34	33.1	153	43.6
Declared extinct	33	31.7	29	20.1	44	42.7	106	30.1
Time	9	8.7	57	39.6	19	18.4	85	24.2
Not Specified	1	1.0	0	0	6	5.8	7	1.9
Total	104		144		103		351	

For 7 species (1 amphibian and 6 mammals) the type of rediscovery was not specified.

The current threat level for rediscovered amphibians, birds, and mammals is several times higher than in all other species in each taxonomic class (Table 2). Moreover, our results suggest that the year of rediscovery and number of years missing weakly predicts whether or not a species is currently threatened (Tables S2 and S3). These analyses suggest that species that disappeared for short periods of time are just as threatened as those missing for many years. Surprisingly, non-threatened bird species were missing for the longest periods of time, however, overall there is no apparent relationship between threat status and the number of years missing (Figure S4).

**Table 2:** The number of rediscovered amphibian, bird, and mammal species in each IUCN Red list category and threat level compared to all other species from each taxonomic class.

	Amphibians		Birds		Mammals	
# of Species (% of Total)	Rediscovered	Other	Rediscovered	Other	Rediscovered	Other
Total	99^a^	6185	143^b^	9853	103	5386
EX	3 (3)	34 (0.5)	3 (2.1)	130 (1.3)	-	76 (1.4)
EW	1 (1)	1 (0)	-	4 (0)	-	2 (0)
CR	42 (42.5)	442 (7.1)	49 (34.3)	143 (1.5)	23 (22.3)	165 (3.1)
EN	13 (13.1)	741 (12)	35 (24.5)	327 (3.3)	30 (29.1)	420 (7.8)
VU	8 (8.1)	649 (10.5)	30 (21.0)	639 (6.5)	16 (15.5)	490 (9.1)
NT	2 (2)	380 (6.1)	11 (7.7)	826 (8.4)	6 (5.8)	314 (5.8)
LC	4 (4)	2367 (38.3)	5 (3.5)	7729 (78.4)	5 (4.9)	3106 (57.7)
DD	26 (26.3)	1571 (25.4)	10 (7.0)	55 (0.6)	23 (22.3)	814 (15.1)
**Schipper's threat level (%)**	**92**	**40**	**86**	**11**	**86**	**23**
**Threat level (range)**	**(68 to 94)**	**(30 to 55)**	**(80 to 87)**	**(11 to 12)**	**(67 to 89)**	**(20 to 35)**

a A total of 104 amphibian species have been rediscovered, but only 99 of them are in the IUCN database. Six species are not recognized taxonomically by the IUCN.

b A total of 144 bird species have been rediscovered, but only 143 of them are in the IUCN database. One species is not recognized taxonomically by the IUCN.

Categories: EX, Extinct; EW, Extinct in the Wild; CR, Critically Endangered; EN, Endangered; VU, Vulnerable; NT, Near threatened; LC, Least Concern; DD, Data Deficient. Schipper's threat level = [(VU+EN+CR)/(Total−DD)]×100 (see [16]). The range is between [(VU+EN+CR)/Total]×100 and [(VU+EN+CR+DD)/Total]×100. Additionally, 3 EX and 1 EW amphibian species are included in the threat level as individuals of each species were rediscovered in the wild.

Approximately 95% of all species rediscovered have restricted ranges (based on <50,000 km^2^ applied by Stattersfield et al. [22] to define Endemic Bird Areas, Figure S5); specifically, 99%, 91%, and 97% of rediscovered threatened amphibian, bird, and mammal species, respectively, are range-restricted. We tested whether species gone missing for long periods of time have smaller ranges compared to species that disappeared for short periods of time using generalized linear models and found no relationship between range size and the number of years a species went missing—rediscovered species are range-restricted independent of the number of years missing (*n* = 309, Tables S4A and B). These trends differed in magnitude between taxonomic groups; however the direction in trends were similar among groups (Table S4B).

## Discussion

Our findings show that a substantial number of species have been rediscovered. We found that 92%, 86% and 86% of all rediscovered amphibians, birds and mammals are currently threatened, respectively. Furthermore, after plotting accumulation curves of threatened and non-threatened species by rediscovery year (see Figure 2), we found that rediscoveries of threatened species increased exponentially with time, whereas the rediscovery of non-threatened species leveled off. This suggests that newly rediscovered species likely have a higher probability of being threatened (inferred from an increasing curve) and a low probability of being non-threatened (inferred from a saturating curve). Perhaps, it may be difficult for species that were highly threatened to recover, or many of these naturally rare species will always meet the criteria for an IUCN-threatened species. In the end, although they are proven to be extant, these species still have the potential to disappear forever.

Rediscoveries for all three taxonomic groups increased with time, even after accounting for the number of taxonomists. The continual increase in rediscoveries per decade may be explained by: 1) the number of species thought by experts to have gone extinct is increasing, therefore bolstering the potential for rediscoveries, and 2) there have been increased expeditions and survey effort supported by institutions and non-profit organizations, particularly in the poorly-known tropics where many rediscovered species have been found. Previous research suggests that moderate search effort is associated with increased mammal rediscoveries even though most rediscovered mammals have not been adequately searched for [9]. Nonetheless, considering the increase in rediscoveries, we are confident that many species presently thought to have gone extinct by experts remain extant, particularly those species that are only known from type specimens. With continued support for biological surveys, particularly in the tropics, many of these species will undoubtedly be relocated with time. The question is however: will these species be relocated before the multitude of human disturbances (e.g., wildlife trade, invasive species, habitat loss, and climate change) drives them to extinction [6]? And, even after their rediscovery, will we be able to adequately protect them?

Our analyses suggest that the majority of species are acutely threatened post-rediscovery; many species rediscovered decades ago are still Critically Endangered, Endangered or Vulnerable [3]. One way to gauge the change in a species' threat status over time would be to retrospectively determine each species' IUCN category at the time of rediscovery and then compare to its current status. In the end, however, we feel the result is the same; approximately 88% of rediscovered species are currently threatened. Thus, regardless of their conservation status at the time of rediscovery, their status has either deteriorated towards or remained at a threatened status. The apparent lack of a relationship between the number of years missing and year of rediscovery and whether or not a species was threatened might be explained by several potential reasons. Lack of conservation efforts may explain why older rediscovered species are just as threatened as newly rediscovered species but their populations could also be so small that a full recovery is very difficult. In the end, there is no guarantee for the long-term survival of a rediscovered species. Thus, even though three amphibians (*Adenomus kandianus*, *Philautus stellatus*, and *P. travancoricus*) considered extinct by the IUCN were recently found (in 2009 and 2010), many rediscovered species remain under serious threat of extinction. For example, there are several rediscovered species that have likely gone extinct from habitat conversion and disease since their rediscovery (e.g., *Zyzomys pedunculatus* disappeared shortly after being rediscovered in 1996 but was recently rediscovered again in 2010 and greater akialoa, *Hemignathus ellisianus* was rediscovered before it disappeared again and is now considered Extinct; [3]). Many of the 351 rediscovered species recorded in this study are likely to go extinct without aggressive conservation efforts.

Perhaps the species most susceptible to extinction post-rediscovery are the 106 species that were considered extinct by researchers prior to their rediscovery. We observed that many of these species were initially considered extinct because researchers witnessed a severe population decline. This however does not mean that future survey efforts should be entirely focused on supposedly extinct species. The majority of rediscoveries comprised species that were so rare or hard-to-find that their only confirmed occurrence was from their initial description. This is expected as the majority of rediscoveries occurred in the understudied tropics [23], [24]. More surveys for missing species are essential for biological conservation [11] and focusing these future search efforts on species only known from type specimens is essential to adequately determine their true threat status [25]. A substantial amount of search effort is likely required to find small ranged species with small populations [26]. Therefore, prioritizing future search efforts among these different types of rediscovery scenarios should be carefully weighed as searching for a species that underwent a population decline may unnecessarily exhaust limited conservation dollars that could be better used if allocated to understudied species (i.e., species only known from type specimens) that have considerable potential to be rediscovered and protected with conservation actions. The formulation of an official list of species that are suspected to be missing, the number of failed surveys, and why they disappeared and for how long, would lead to more successful search efforts and subsequent conservation (see [7], [27]). Attempts to create such lists have already been made for birds; there are some 20 species that are considered “missing” from the Neotropics [28] and 14 “missing” bird species awaiting rediscovery in Asia [7], some of which have not been seen for over 150 years [28]. According to our study, focusing conservation efforts on rare species that have gone unseen for extended periods of time should prove fruitful in relocating “missing” species. Once found, conservation actions may bolster the long-term survival of these rediscovered species (e.g., as was done with the Cebu flowerpecker *Dicaeum quadricolor* post-rediscovery in 1996 and the Gurney's pitta *Pitta gurneyi* in 1986).

It is important to note that we quantified our different rediscovery types based on expert opinion. In other words our values for “declared extinct” are that of an opinion by professionals in the field of study and not that defined by organizations such as IUCN. Increased rigor in listing procedures is paramount for proper conservation. For example, the Cebu flowerpecker was considered extinct for almost 40 years before being rediscovered in 1996. Under the presumption that the species was extinct, few surveys were conducted to document the species' existence and as a result no conservation actions were in place [29]. This allowed for the last remaining tracts of suitable forest to be further degraded [29]. Only after its rediscovery was suitable conservation allocated towards protecting this species. Thus, caution must be used when officially declaring a species extinct. One way organizations, such as the IUCN, have attempted to minimize listing mistakes that result in “romeo error” (i.e., whereby we abandon conservation of a species based on the assumption that it is extinct when in fact it may still be extant) is by creating more rigorous listing procedures [29]. An additional means to alleviate false listing of extinct species was developed by Butchart et al. [27], which created the “Possibly Extinct” criterion within the Critically Endangered category to identify those species for which there is reasonable, but not complete, evidence that they may be gone forever. This marker (“Possibly Extinct”) is now incorporated into the IUCN system.

Many rediscovered species appear to be naturally hard-to-find or understudied; 153 (43.6%) of the rediscoveries were the first record since the description of the species. This may result from limited survey effort within a species' geographic range (although many references stated that extensive surveys were conducted) [26]. Nonetheless, the apparent rarity of these species may also be a product of their range size. We found no relationship between range size and the number of years a species went missing or when it was rediscovered. Instead, the majority of rediscovered species, new and old, have small isolated populations. Previous work by Fisher and Blomberg [9] suggested that species most likely to be rediscovered are those with large ranges that declined from habitat loss. Although we did not account for exact mechanisms that caused population declines, we found that the majority (approximately 95%) of rediscovered species in our study have restricted ranges (<50,000 km^2^). We recognize that disease (e.g., chytrid fungus), body size, and other threats all contribute to the disappearance of a species as extinctions are commonly caused by multiple synergistic threats [2]. Small geographical range size is the main predictor of extinction threat in terrestrial vertebrates [19], [30], particularly when species are located in areas with high habitat conversion [9], [31]. Thus, the rediscovered species identified in this study are likely vulnerable to extinction if disturbances persist within their restricted ranges.

Rediscovered birds and mammals occurred at variable elevations whereas most rediscovered amphibian species were endemic to high elevations (for example, the mean minimum elevation of occurrence for amphibians was 1199 m; Figure S3). This may be problematic as countries that appear to have the greatest amount of remaining high elevation forests are losing it the fastest [32]. Additionally, the cooler temperatures typical at high elevations make many amphibian species more susceptible to chytrid fungus, a major threat and cause of decline to many of the amphibians (especially stream-breeding species) identified in this study [33]. When comparing historical distributions of rediscovered mammals to current distributions, Fisher [31] found an up-slope shift of 35% between last recorded locations (*c.* 520 m) and rediscovery sites (*c.* 700 m) of mammals. More importantly, Fisher [31] found fewer than 5% of rediscovered mammals were located at their original location of disappearance. Thus future search effort for missing species should not be restricted entirely to the region of their last occurrence.

In addition to location, range size, and elevation, there are many other variables, not included in this study, that may influence when and whether or not a species is rediscovered. For example 1) smaller mammals went missing for longer periods of time than larger more charismatic species [15], 2) a species' behavior (e.g., diurnal versus nocturnal habits) may affect whether or not it is rediscovered by field researchers, 3) political instability and government restrictions may influence whether or not researchers have access to search for missing species [34] and 4) areas with minimal human disturbance are predicted to have higher proportions of undescribed species [35] and thus these understudied areas may also harbor a large number of missing species.

The loss and rediscovery of a species can at times be controversial, particularly when substantial conservation dollars are spent to conserve rediscovered species that appear to be extant, even though proof of the rediscovery is unconfirmed (e.g., Ivory-billed Woodpecker, *C. principalis*) [14], [36]. On the other hand, public support for conservation can be lost when lands remain protected for a species that have disappeared; this is particularly true when these lands can instead be used to improve community livelihoods [37], [38]. In some cases, reserves may become declassified as they are considered “over-protected” [38]. As human-wildlife conflicts worsen and useable land diminishes [39], conservation of protected areas will become more challenging [40] as demands by local communities to deregulate protected areas established for protecting threatened species will likely become increasingly common. Range-restricted, highly threatened species may remain undetected for many years—mean of 61 years in our study— making conservation planning difficult. Therefore, extensive biodiversity surveys should be an integral part of conservation initiatives [11].

Overall, considering the array of negative synergies that are driving species losses [2], many of the critically threatened species that have been rediscovered will remain on the brink of oblivion.

## Supporting Information

Figure S1 **The number of taxonomists describing species and species rediscovered per year.** The dotted line represents the number of taxonomists describing species in a given year; the bar chart represents the number of species rediscovered per year.
(TIF)

Figure S2 **Number of species rediscovered per year divided by the number of taxonomists.** The number of species rediscovered per year divided by the number of taxonomists who were actively describing species in the same year (black lines). The colored circles represent the number of species rediscovered per 10-year period divided by the number of taxonomist describing species during the same time period.
(TIF)

Figure S3 **Minimum elevation distribution for all rediscovered amphibian, bird, and mammal species. Circles = outliers.**
(TIF)

Figure S4 **Number of years missing plotted against IUCN status for rediscovered species in each taxonomic class.** The mean number of years missing plotted against IUCN status for rediscovered species in each taxonomic class. Circles represent outliers.
(TIF)

Figure S5 **Range size distribution for all rediscovered amphibian, bird, and mammal species. Circles = outliers.**
(TIF)

Table S1 **List of amphibian, bird, and mammal species rediscovered.** Also shown are the year last seen, year rediscovered, number of years gone missing, the 2009 IUCN Red List conservation status and type of rediscovery.
(DOC)

Table S2 **Binomial GLMs were used to investigate the relationship between year of rediscovery and years gone missing and whether or not a species was threatened according to IUCN Red List status.** Predictor terms shown are year rediscovered and total number of years gone missing. We used a binomial response coded as threatened or non-threatened. Also shown are the number of parameters (*k*), log likelihood (*LL*), the difference in AIC*_c_* of each model from the highest ranked model (ΔAIC*_c_*), AIC*_c_* weights representing the probability of each model being the best (*w*AIC*_c_*), and the percent deviance explained by each model (%DE). Models are ranked by AIC*_c_* weights.
(DOC)

Table S3 **The estimate, standard error (SE), z-value, and p-value for each parameter included in each model.** The binomial GLMs used threatened or non-threatened as the response variable and year rediscovered and number of years gone missing as predictor variables.
(DOC)

Table S4 **Rediscovered species are highly range-restricted independent of the years gone missing.** Generalized linear models were used to investigate the relationship between species range size and the number of years a species went missing (Table A). The models are ranked by Akaike's Information Criterion corrected for small sample size (AIC*_c_*). Predictor terms shown in Table A are Year = number of years missing and class (i.e., amphibian, bird, or mammal) as a fixed effect. Also shown are the number of parameters (*k*), log likelihood (*LL*), the difference in AIC*_c_* of each model from the highest ranked model (ΔAIC*_c_*), AIC*_c_* weights representing the probability of each model being the best (*w*AIC*_c_*), and the percent deviance explained by each model (%DE). Table B provides the estimate, standard error (SE), z-value, and p-value for each parameter included in each model.
(DOC)
