# Supplementary figures and images for: The World's Rediscovered Species: Back from the Brink?
Source: PLoS One. 2011 Jul 27;6(7):e22531. doi: 10.1371/journal.pone.0022531 (PMC3144889; doi:10.1371/journal.pone.0022531)

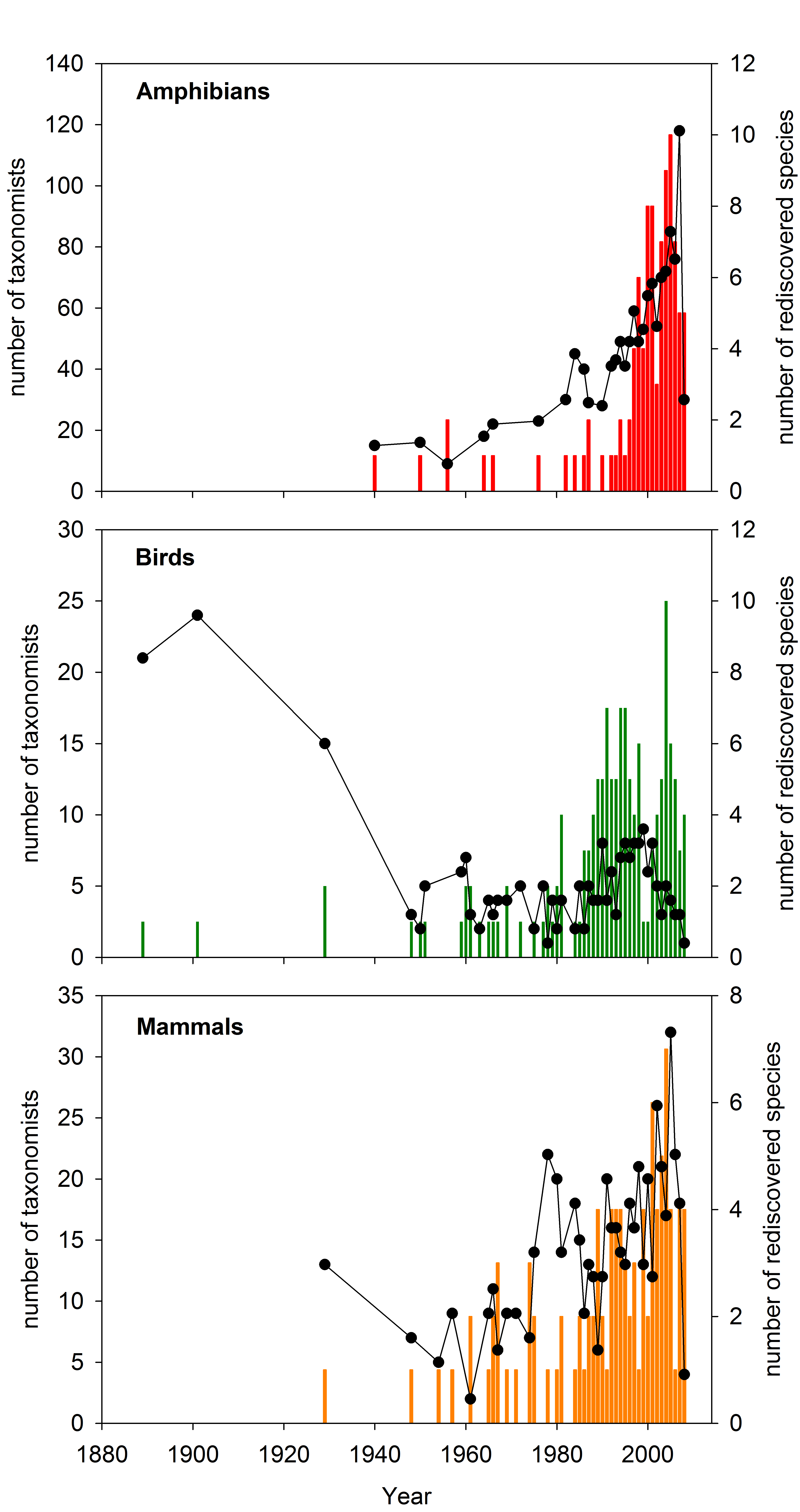

Supplement: Figure S1 — The number of taxonomists describing species and species rediscovered per year. The dotted line represents the number of taxonomists describing species in a given year; the bar chart represents the number of species rediscovered per year. (TIF) [file pone.0022531.s001.tif]

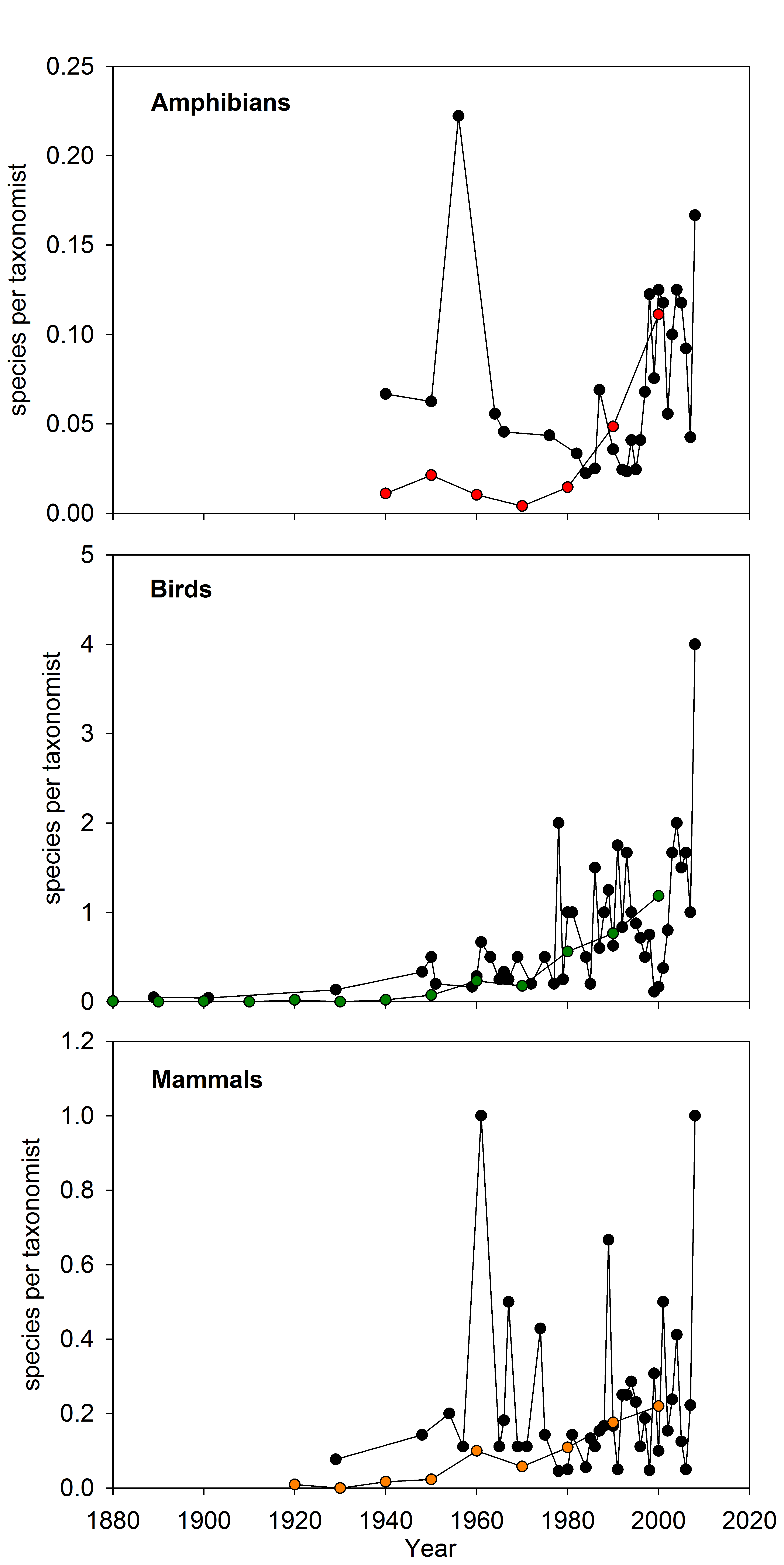

Supplement: Figure S2 — Number of species rediscovered per year divided by the number of taxonomists. The number of species rediscovered per year divided by the number of taxonomists who were actively describing species in the same year (black lines). The colored circles represent the number of species rediscovered per 10-year period divided by the number of taxonomist describing species during the same time period. (TIF) [file pone.0022531.s002.tif]
