# Supplementary material for: The World's Rediscovered Species: Back from the Brink?
Source: PLoS One. 2011 Jul 27;6(7):e22531. doi: 10.1371/journal.pone.0022531 (PMC3144889; doi:10.1371/journal.pone.0022531)
Supplement: Table S1 — List of amphibian, bird, and mammal species rediscovered. Also shown are the year last seen, year rediscovered, number of years gone missing, the 2009 IUCN Red List conservation status and type of rediscovery. (DOC) [file pone.0022531.s006.doc]

**Table S1.** List of amphibian, bird, and mammal species rediscovered. Also shown are the year last seen, year rediscovered, number of years gone missing, the 2009 IUCN Red List conservation status and type of rediscovery. NA indicates that data are unavailable.

| **Name** | **Last Seen** | **Rediscovered** | **Years Missing** | **IUCN Status** | **Type** |
| --- | --- | --- | --- | --- | --- |
| **Amphibians** |  |  |  |  |  |
| *Adenomus kandianus* | 1872 | 2009 | 137 | EX | holotype |
| *Allobates humilis* | 1980 | 2002 | 22 | VU | holotype |
| *Alsodes montanus* | 1902 | 1964 | 62 | CR | holotype |
| *Ameerega boliviana* | 1902 | 1998 | 96 | LC | holotype |
| *Ameerega planipaleae* | 1996 | 2007 | 11 | CR | holotype |
| *Anaxyrus baxteri* | 1980 | 1987 | 7 | EW | extinct |
| *Ansonia ornata* | 1890 | 1990 | 100 | EN | holotype |
| *Atelopus bomolochos* | 1991 | 2002 | 11 | CR | extinct |
| *Atelopus carrikeri* | 1994 | 2008 | 14 | CR | extinct |
| *Atelopus cruciger* | 1982 | 2003 | 21 | CR | extinct |
| *Atelopus ebenoides* | 1995 | 2006 | 11 | CR | extinct |
| *Atelopus eusebianus* | 1991 | 2004 | 13 | CR | extinct |
| *Atelopus laetissimus* | 1992 | 2006 | 14 | CR | extinct |
| *Atelopus mucubajiensis* | 1994 | 2004 | 10 | CR | extinct |
| *Atelopus nahumae* | 1992 | 2006 | 14 | CR | extinct |
| *Atelopus palmatus* | 1937 | 2008 | 71 | DD | extinct |
| *Atelopus seminiferus* | 1874 | 2001 | 127 | CR | holotype |
| *Atelopus varius* | 1996 | 2003 | 7 | CR | holotype |
| *Austrochaperina brevipes* | 1896 | 1986 | 90 | DD | holotype |
| *Austrochaperina yelaensis* | 1960 | 2004 | 44 | DD | holotype |
| *Barbourula busuangensis* | 1923 | 1940 | 17 | VU | time |
| *Barbourula kalimantanensis* | 1995 | 2007 | 12 | EN | time |
| *Bolitoglossa capitana* | 1971 | 2005 | 34 | CR | holotype |
| *Boophis williamsi* | 1979 | 2001 | 22 | CR | extinct |
| *Bradytriton silus* | 1976 | 2009 | 33 | CR | extinct |
| *Bufo sumatranus* | 1860 | 2001 | 141 | CR | holotype |
| *Calluella volzi* | 1905 | 1999 | 94 | DD | extinct |
| *Cardioglossa aureoli* | 1963 | 2003 | 40 | EN | holotype |
| *Chiromantis simus* | 1911 | 1999 | 88 | LC | extinct |
| *Chiropterotriton magnipes* | NA | 2005 | NA | CR | extinct |
| *Conraua derooi* | 1980 | 2005 | 25 | CR | holotype |
| *Craugastor gulosus* | 1875 | 1976 | 101 | EN | extinct |
| *Craugastor milesi* | 1992 | 2009 | 17 | CR | holotype |
| *Dendropsophus ruschii* | 1987 | 2005 | 18 | DD | holotype |
| *Dendrotriton cuchumatanus* | 1975 | 2005 | 30 | CR | holotype |
| *Eleutherodactylus cubanus* | 1936 | 1994 | 58 | CR | holotype |
| *Eleutherodactylus turquinensis* | 1936 | 1994 | 58 | CR | holotype |
| *Excidobates captivus* | 1929 | 2006 | 77 | DD | holotype |
| *Excidobates mysteriosus* | 1929 | 1982 | 53 | EN | holotype |
| *Gastrotheca splendens* | 1857 | 1997 | 140 | EN | holotype |
| *Gegeneophis seshachari* | 1967 | 2003 | 36 | DD | time |
| *Holoaden luederwaldti* | 1967 | 2005 | 38 | DD | holotype |
| *Hyalinobatrachium talamancae* | 1952 | 2001 | 49 | LC | holotype |
| *Hylarana igorota* | 1920 | 2001 | 81 | VU | holotype |
| *Hyloscirtus charazani* | 1970 | 1997 | 27 | EN | holotype |
| *Hyophryne histrio* | 1954 | 2000 | 46 | DD | holotype |
| *Hypsiboas melanopleura* | 1912 | 2004 | 92 | DD | holotype |
| *Ichthyophis hypocyaneus* | 1827 | 2000 | 173 | DD | extinct |
| *Incilius cristatus* | NA | 1998 | NA | CR | extinct |
| *Isthmohyla rivularis* | 1988 | 2007 | 19 | CR | holotype |
| *Ixalus lateralis* | 1871 | 2008 | 137 | NA | extinct |
| *Kurixalus naso* | 1912 | 2000 | 88 | DD | extinct |
| *Lechriodus papuanus* | 1927 | 1956 | 29 | NA | extinct |
| *Lithobates onca* | 1950 | 1995 | 45 | EN | extinct |
| *Lithobates sevosus* | NA | 1987 | NA | CR | extinct |
| *Lithobates vibicarius* | 1990 | 2002 | 12 | CR | holotype |
| *Litoria aurea* | 1981 | 1999 | 18 | VU | time |
| *Litoria castanea* | 1975 | 2010 | 35 | CR | extinct |
| *Litoria dorsalis* | 1877 | 1956 | 79 | LC | extinct |
| *Litoria leucova* | NA | 1993 | NA | DD | holotype |
| *Litoria lorica* | 1991 | 2008 | 17 | CR | holotype |
| *Liuixalus romeri* | 1953 | 1984 | 31 | EN | holotype |
| *Mantella manery* | 1999 | 2004 | 5 | VU | holotype |
| *Mantidactylus pauliani* | 1979 | 2001 | 22 | CR | holotype |
| *Melanobatrachus indicus* | 1878 | 1997 | 119 | EN | not specified |
| *Melanophryniscus macrogranulosus* | 1973 | 2004 | 31 | VU | extinct |
| *Melanophryniscus pachyrhynus* | 1905 | 2005 | 100 | DD | holotype |
| *Micrixalus kottigeharensis* | 1937 | 2001 | 64 | CR | holotype |
| *Nanorana fansipani* | 1938 | 2000 | 62 | DD | holotype |
| *Nimbaphrynoides liberiensis* | 1978 | 2010 | 32 | CR | holotype |
| *Nyctixalus margaritifer* | NA | 1997 | NA | VU | holotype |
| *Oreobates granulosus* | 1903 | 2008 | 105 | NA | holotype |
| *Oreobates heterodactylus* | 1937 | 2005 | 68 | DD | holotype |
| *Oreophryne insulana* | NA | 1998 | NA | DD | time |
| *Oscaecilia osae* | 1990 | 2004 | 14 | DD | holotype |
| *Paratelmatobius gaigeae* | 1931 | 2003 | 72 | DD | time |
| *Paratelmatobius mantiqueira* | 1953 | 2005 | 52 | DD | holotype |
| *Peltophryne lemur* | 1930 | 1966 | 36 | CR | holotype |
| *Philautus chalazodes* | 1878 | 2003 | 125 | CR | holotype |
| *Philautus stellatus* | 1853 | 2009 | 156 | EX | extinct |
| *Philautus travancoricus* | 1910 | 2010 | 100 | EX | holotype |
| *Phrynopus peruanus* | 1874 | 2005 | 131 | DD | holotype |
| *Phyllodytes wuchereri* | 1873 | 2004 | 131 | DD | holotype |
| *Pingia granulosus* | 1933 | 2009 | 76 | NA | holotype |
| *Platymantis cornutus* | 1922 | 1998 | 76 | VU | holotype |
| *Platymantis hazelae* | 1922 | 1998 | 76 | EN | holotype |
| *Plethodon neomexicanus* | 1913 | 1950 | 37 | NT | holotype |
| *Protohynobius puxiongensis* | 1965 | 2009 | 44 | DD | holotype |
| *Pseudoeurycea nigra* | NA | 2000 | NA | CR | holotype |
| *Pseudoeurycea nigromaculata* | 1985 | 2006 | 21 | CR | extinct |
| *Pseudoeurycea parva* | 1985 | 2007 | 22 | CR | time |
| *Rhacophorus bifasciatus* | 1926 | 2006 | 80 | NT | extinct |
| *Rhacophorus lateralis* | 1883 | 2000 | 117 | EN | holotype |
| *Rhacophorus poecilonotus* | 1920 | 2000 | 80 | DD | holotype |
| *Rhinella boulengeri* | 1936 | 2000 | 64 | DD | holotype |
| *Rhinella leptoscelis* | 1912 | 2006 | 94 | NA | holotype |
| *Sphaenorhynchus pauloalvini* | 1973 | 2007 | 34 | DD | holotype |
| *Taudactylus acutirostris* | 1857 | 1996 | 139 | CR | extinct |
| *Taudactylus eungellensis* | 1987 | 1992 | 5 | CR | time |
| *Taudactylus rheophilus* | 1991 | 1996 | 5 | CR | extinct |
| *Telmatobius gigas* | 1966 | 1998 | 32 | CR | holotype |
| *Telmatobufo venustus* | 1899 | 1999 | 100 | EN | extinct |
| *Thorius magnipes* | 1983 | 2003 | 20 | CR | time |
| *Thorius minutissimus* | 1949 | 2001 | 52 | CR | extinct |
| **Birds** |  |  |  |  |  |
| *Acrocephalus orinus* | 1867 | 2006 | 139 | DD | holotype |
| *Actenoides bougainvillei* | 1953 | 1994 | 41 | VU | time |
| *Aegotheles savesi* | 1880 | 1998 | 118 | CR | holotype |
| *Aegotheles tatei* | 1969 | 2003 | 34 | DD | holotype |
| *Aepypodius bruijnii* | 1938 | 2002 | 64 | EN | time |
| *Amaurocichla bocagei* | 1928 | 1990 | 62 | VU | time |
| *Amaurospiza carrizalensis* | 2003 | 2008 | 5 | CR | time |
| *Amazilia castaneiventris* | 1979 | 2004 | 25 | EN | time |
| *Amazilia luciae* | 1950 | 1988 | 38 | CR | time |
| *Amblyornis flavifrons* | 1895 | 1981 | 86 | LC | holotype |
| *Anas nesiotis* | 1886 | 1975 | 89 | CR | extinct |
| *Anodorhynchus leari* | 1856 | 1978 | 122 | EN | holotype |
| *Aplonis pelzelni* | 1956 | 1995 | 39 | CR | extince |
| *Arborophila davidi* | 1927 | 1991 | 64 | NT | holotype |
| *Atlapetes pallidiceps* | 1969 | 1998 | 29 | CR | extinct |
| *Atrichornis clamosus* | 1889 | 1961 | 72 | VU | extinct |
| *Aythya innotata* | 1991 | 2006 | 15 | CR | extinct |
| *Bangsia melanochlamys* | 1948 | 1999 | 51 | VU | time |
| *Bostrychia bocagei* | 1920 | 1990 | 70 | CR | time |
| *Bradypterus seebohmi* | 1894 | 2004 | 110 | LC | holotype |
| *Cacicus koepckeae* | 1965 | 1998 | 33 | VU | holotype |
| *Calyptura cristata* | 1890 | 1996 | 106 | CR | time |
| *Campephilus principalis* | 1944 | 2004 | 60 | CR | extinct |
| *Caprimulgus noctitherus* | 1911 | 1961 | 50 | CR | extinct |
| *Caprimulgus solala* | 1989 | 2009 | 20 | VU | holotype |
| *Carpococcyx viridis* | 1916 | 1997 | 81 | CR | time |
| *Celeus obrieni* | 1926 | 2006 | 80 | CR | holotype |
| *Chlorospingus flavovirens* | 1935 | 1972 | 37 | VU | holotype |
| *Chondrohierax wilsonii* | 2001 | 2009 | 8 | CR | time |
| *Cisticola melanurus* | 1972 | 2005 | 33 | DD | time |
| *Clytoctantes alixii* | 1965 | 2004 | 39 | EN | time |
| *Clytoctantes atrogularis* | 1986 | 2004 | 18 | VU | time |
| *Clytorhynchus sanctaecrucis* | 1927 | 2004 | 77 | EN | holotype |
| *Coeligena orina* | 1951 | 2004 | 53 | CR | holotype |
| *Colluricincla sanghirensis* | 1881 | 1995 | 114 | CR | holotype |
| *Columba argentina* | 1931 | 2008 | 77 | CR | time |
| *Conothraupis mesoleuca* | 1938 | 2003 | 65 | CR | holotype |
| *Copsychus cebuensis* | 1965 | 1981 | 16 | EN | time |
| *Corvus unicolor* | 1900 | 2007 | 107 | CR | holotype |
| *Cossypha heinrichi* | 1957 | 2005 | 48 | VU | time |
| *Crocias langbianis* | 1938 | 1994 | 56 | EN | holotype |
| *Dicaeum quadricolor* | 1906 | 1992 | 86 | CR | extinct |
| *Diglossa gloriosissima* | 1965 | 2003 | 38 | EN | time |
| *Dysithamnus occidentalis* | 1941 | 1991 | 50 | VU | time |
| *Eleoscytalopus psychopompus* | 1983 | 2004 | 21 | CR | extinct |
| *Embernagra longicauda* | 1918 | 1928 | 10 | NT | holotype |
| *Eriocnemis mirabilis* | 1987 | 1997 | 10 | CR | time |
| *Estrilda poliopareia* | 1987 | 2002 | 15 | VU | extinct |
| *Eurostopodus diabolicus* | 1931 | 1996 | 65 | VU | holotype |
| *Eutrichomyias rowleyi* | 1878 | 1998 | 120 | CR | holotype |
| *Eutriorchis astur* | 1950 | 1993 | 43 | EN | extinct |
| *Ficedula bonthaina* | 1935 | 1995 | 60 | EN | time |
| *Ficedula disposita* | 1967 | 1991 | 24 | NT | holotype |
| *Ficedula henrici* | 1898 | 2001 | 103 | NT | time |
| *Formicarius rufifrons* | 1955 | 1995 | 40 | NT | time |
| *Formicivora erythronotos* | 1850 | 1987 | 137 | EN | time |
| *Gallicolumba hoedtii* | 1898 | 2008 | 110 | EN | time |
| *Garrulax bieti* | 1989 | 2008 | 19 | VU | time |
| *Garrulax courtoisi* | 1919 | 2000 | 81 | CR | holotype |
| *Grallaria alleni* | 1979 | 1990 | 11 | VU | extinct |
| *Grallaria milleri* | 1942 | 1994 | 52 | EN | holotype |
| *Hapalopsittaca fuertesi* | 1911 | 2002 | 91 | CR | holotype |
| *Hemignathus ellisianus* | 1895 | 1960 | 65 | EX | extinct |
| *Hemitriccus inornatus* | 1831 | 1992 | 161 | LC | holotype |
| *Hemitriccus kaempferi* | 1929 | 1991 | 62 | EN | time |
| *Heteroglaux blewitti* | 1884 | 1997 | 113 | CR | extinct |
| *Hylopezus auricularis* | 1937 | 1994 | 57 | VU | holotype |
| *Laniarius brauni* | 1957 | 2005 | 48 | EN | time |
| *Lanius newtoni* | 1928 | 1990 | 62 | CR | time |
| *Lepidothrix vilasboasi* | 1957 | 2002 | 45 | VU | holotype |
| *Leptodon forbesi* | 1922 | 1987 | 65 | CR | holotype |
| *Loddigesia mirabilis* | 1835 | 1965 | 130 | EN | time |
| *Lophura edwardsi* | 1928 | 1996 | 68 | EN | extinct |
| *Lophura inornata* | 1950 | 1985 | 35 | VU | time |
| *Luscinia ruficeps* | 1905 | 1963 | 58 | VU | holotype |
| *Macroagelaius subalaris* | 1953 | 2005 | 52 | EN | time |
| *Madanga ruficollis* | 1923 | 1995 | 72 | EN | holotype |
| *Malaconotus kupeensis* | 1951 | 1989 | 38 | EN | holotype |
| *Melanocharis arfakiana* | 1933 | 1992 | 59 | DD | time |
| *Melanospiza richardsoni* | 1886 | 1929 | 43 | EN | holotype |
| *Merulaxis stresemanni* | 1945 | 1995 | 50 | CR | time |
| *Moho bishopi* | 1904 | 1981 | 77 | EX | time |
| *Moho braccatus* | 1945 | 1960 | 15 | EX | extinct |
| *Monarcha boanensis* | 1918 | 1991 | 73 | CR | holotype |
| *Monarcha julianae* | 1955 | 1986 | 31 | DD | holotype |
| *Myrmotherula fluminensis* | 1988 | 1994 | 6 | CR | holotype |
| *Nemosia rourei* | 1941 | 1998 | 57 | CR | time |
| *Neospiza concolor* | 1888 | 1991 | 103 | CR | holotype |
| *Newtonia fanovanae* | 1933 | 1989 | 56 | VU | holotype |
| *Nipponia nippon* | 1978 | 1981 | 3 | EN | time |
| *Nyctibius leucopterus* | 1817 | 1993 | 176 | LC | holotype |
| *Oceanites maorianus* | 1850 | 2003 | 153 | CR | holotype |
| *Ognorhynchus icterotis* | 1919 | 2005 | 86 | CR | time |
| *Otus alfredi* | 1897 | 1994 | 97 | EN | holotype |
| *Otus capnodes* | 1886 | 1992 | 106 | CR | extinct |
| *Otus insularis* | 1906 | 1959 | 53 | EN | extinct |
| *Otus pauliani* | 1886 | 1989 | 103 | CR | time |
| *Parotia berlepschi* | 1981 | 2005 | 24 | NA | holotype |
| *Penelope albipennis* | 1876 | 1977 | 101 | CR | holotype |
| *Perdicula manipurensis* | 1932 | 2006 | 74 | VU | time |
| *Pezoporus occidentalis* | 1912 | 1979 | 67 | CR | extinct |
| *Phodilus prigoginei* | 1951 | 1996 | 45 | EN | holotype |
| *Pithys castaneus* | 1937 | 2001 | 64 | NT | holotype |
| *Pitta gurneyi* | 1952 | 1986 | 34 | EN | extinct |
| *Pitta schneideri* | 1918 | 1988 | 70 | VU | time |
| *Pitta venusta* | 1918 | 1988 | 70 | VU | time |
| *Ploceus aureonucha* | 1993 | 2006 | 13 | EN | time |
| *Ploceus megarhynchus* | 1869 | 1889 | 20 | VU | holotype |
| *Ploceus ruweti* | 1960 | 2009 | 49 | DD | holotype |
| *Poecilotriccus senex* | 1830 | 1993 | 163 | LC | holotype |
| *Porphyrio hochstetteri* | 1898 | 1948 | 50 | EN | extinct |
| *Pseudobulweria becki* | 1929 | 2007 | 78 | CR | holotype |
| *Pseudobulweria macgillivrayi* | 1855 | 1984 | 129 | CR | holotype |
| *Pseudocalyptomena graueri* | 1909 | 1929 | 20 | VU | time |
| *Pseudonestor xanthophrys* | 1895 | 1950 | 55 | CR | extinct |
| *Pterodroma cahow* | 1620 | 1951 | 331 | EN | extinct |
| *Pterodroma madeira* | 1965 | 1969 | 4 | EN | extinct |
| *Pterodroma magentae* | 1869 | 1978 | 109 | CR | extinct |
| *Pycnonotus nieuwenhuisii* | 1937 | 1992 | 55 | DD | time |
| *Pyrrhula murina* | 1932 | 1967 | 35 | CR | time |
| *Rallus antarcticus* | 1959 | 1998 | 39 | VU | time |
| *Rhinomyias additus* | 1925 | 1995 | 70 | NT | time |
| *Rhinoptilus bitorquatus* | 1848 | 1986 | 138 | CR | extinct |
| *Rhipidura malaitae* | 1930 | 1990 | 60 | VU | time |
| *Sarothrura watersi* | 1930 | 1987 | 57 | EN | holotype |
| *Serinus flavigula* | 1886 | 1989 | 103 | EN | time |
| *Simoxenops striatus* | 1935 | 1989 | 54 | NT | holotype |
| *Siphonorhis brewsteri* | 1928 | 1969 | 41 | NT | extinct |
| *Spelaeornis badeigularis* | 1947 | 2004 | 57 | VU | holotype |
| *Stachyris herberti* | 1920 | 1994 | 74 | NT | holotype |
| *Sterna bernsteini* | 1991 | 2001 | 10 | CR | extinct |
| *Synallaxis kollari* | 1832 | 1966 | 134 | EN | holotype |
| *Tanygnathus gramineus* | 1922 | 1980 | 58 | VU | holotype |
| *Thamnophilus praecox* | 1937 | 1991 | 54 | NT | holotype |
| *Thaumatibis gigantea* | 1962 | 1993 | 31 | CR | time |
| *Tijuca condita* | 1942 | 1980 | 38 | VU | holotype |
| *Toxostoma guttatum* | 1995 | 2004 | 9 | CR | time |
| *Trichocichla rufa* | 1894 | 2003 | 109 | EN | time |
| *Turnix worcesteri* | 1902 | 2009 | 107 | DD | extinct |
| *Tyto sororcula* | 1923 | 1996 | 73 | DD | time |
| *Tyto soumagnei* | 1973 | 1993 | 20 | VU | time |
| *Vermivora bachmanii* | 1833 | 1901 | 68 | CR | holotype |
| *Xenoglaux loweryi* | 1976 | 2007 | 31 | EN | time |
| *Zosterops meeki* | 1990 | 2004 | 14 | DD | time |
| **Mammals** |  |  |  |  |  |
| *Acerodon humilis* | 1899 | 1999 | 100 | EN | extinct |
| *Acomys nesiotes* | 1980 | 2007 | 27 | DD | time |
| *Allocebus trichotis* | 1875 | 1989 | 114 | DD | holotype |
| *Arctocephalus philippii* | 1900 | 1965 | 65 | NT | extinct |
| *Arctocephalus townsendi* | 1850 | 1954 | 104 | NT | extinct |
| *Balantiopteryx infusca* | 1891 | 1991 | 100 | EN | holotype |
| *Brachytarsomys villosa* | 1962 | 2001 | 39 | EN | holotype |
| *Bunolagus monticularis* | 1902 | 1929 | 27 | CR | holotype |
| *Carpomys melanurus* | 1896 | 2008 | 112 | DD | extinct |
| *Cebus flavius* | 1774 | 2006 | 232 | CR | holotype |
| *Cercopithecus sclateri* | NA | 1988 | NA | VU | extinct |
| *Chacodelphys formosa* | 1920 | 2004 | 84 | VU | holotype |
| *Chaetomys subspinosus* | NA | 1989 | NA | VU | not specified |
| *Cheirogaleus sibreei* | 1894 | 2001 | 107 | DD | not specified |
| *Chinchilla chinchilla* | 1953 | 2001 | 48 | CR | extinct |
| *Chinchilla lanigera* | 1955 | 1978 | 23 | CR | extinct |
| *Chodsigoa sodalis* | 1913 | 1997 | 84 | DD | holotype |
| *Crocidura andamanensis* | 1902 | 1999 | 97 | CR | holotype |
| *Crocidura caliginea* | NA | 1990 | NA | LC | time |
| *Crocidura dhofarensis* | 1977 | 2005 | 28 | DD | time |
| *Crocidura jenkinsi* | 1978 | 1999 | 21 | CR | holotype |
| *Crocidura macmillani* | 1915 | 2002 | 87 | VU | holotype |
| *Crocidura picea* | 1940 | 1999 | 59 | EN | holotype |
| *Crocidura trichura* | 1908 | 1985 | 77 | CR | extinct |
| *Cryptochloris zyli* | 1938 | 2003 | 65 | EN | holotype |
| *Cryptotis endersi* | 1941 | 1980 | 39 | EN | holotype |
| *Cryptotis nelsoni* | 1900 | 2009 | 109 | CR | extinct |
| *Dactylopsila tatei* | 1932 | 1992 | 60 | EN | time |
| *Diplogale hosei* | 1955 | 2005 | 50 | VU | time |
| *Dipodomys insularis* | 1989 | 2005 | 16 | CR | extinct |
| *Dobsonia chapmani* | 1975 | 2001 | 26 | CR | extinct |
| *Ectophylla alba* | 1885 | 1961 | 76 | NT | holotype |
| *Eupetaurus cinereus* | 1924 | 1994 | 70 | DD | extinct |
| *Funisciurus duchaillui* | 1952 | 1993 | 41 | DD | holotype |
| *Geocapromys ingrahami* | NA | 1966 | NA | VU | extinct |
| *Glischropus javanus* | 1939 | 2004 | 65 | DD | time |
| *Gymnobelideus leadbeateri* | 1910 | 1961 | 51 | EN | time |
| *Harpiola grisea* | 1872 | 2002 | 130 | DD | holotype |
| *Hipposideros coronatus* | 1871 | 2002 | 131 | DD | holotype |
| *Hipposideros marisae* | 1989 | 2009 | 20 | VU | time |
| *Hipposideros ridleyi* | 1910 | 1974 | 64 | VU | time |
| *Kerivoula africana* | NA | 2000 | NA | EN | time |
| *Latidens salimalii* | 1948 | 1993 | 45 | EN | holotype |
| *Leptomys signatus* | 1936 | 2003 | 67 | LC | holotype |
| *Macropus parma* | 1932 | 1966 | 34 | NT | extinct |
| *Marmosa andersoni* | 1954 | 1997 | 43 | DD | holotype |
| *Mesocapromys angelcabrerai* | 1979 | 2004 | 25 | EN | extinct |
| *Microakodontomys transitorius* | 1993 | 2003 | 10 | VU | holotype |
| *Microcebus myoxinus* | 1893 | 1993 | 100 | DD | extinct |
| *Microtus bavaricus* | 1976 | 2004 | 28 | CR | extinct |
| *Muntiacus montanus* | 1930 | 2008 | 78 | DD | extinct |
| *Muntiacus rooseveltorum* | 1929 | 1994 | 65 | DD | extinct |
| *Mustela nigripes* | 1975 | 1981 | 6 | EN | extinct |
| *Myotis planiceps* | 1970 | 2004 | 34 | EN | extinct |
| *Natalus primus* | 1919 | 1992 | 73 | CR | extinct |
| *Neopteryx frosti* | 1939 | 1985 | 46 | EN | holotype |
| *Nesolagus netscheri* | 1972 | 2000 | 28 | VU | not specified |
| *Nesoromys ceramicus* | 1920 | 1987 | 67 | EN | holotype |
| *Nesoryzomys fernandinae* | 1979 | 1995 | 16 | VU | time |
| *Nesoryzomys swarthi* | 1906 | 1997 | 91 | VU | extinct |
| *Nomascus nasutus* | 1965 | 2002 | 37 | CR | extinct |
| *Nyctimene malaitensis* | 1968 | 2004 | 36 | DD | not specified |
| *Onychogalea fraenata* | 1944 | 1974 | 30 | EN | extinct |
| *Oreonax flavicauda* | 1926 | 1974 | 48 | CR | extinct |
| *Oryzomys gorgasi* | 1971 | 2001 | 30 | EN | holotype |
| *Otomops formosus* | 1939 | 1990 | 51 | DD | holotype |
| *Parantechinus apicalis* | 1884 | 1967 | 83 | EN | extinct |
| *Pardofelis badia* | 1928 | 1992 | 64 | EN | time |
| *Petaurus gracilis* | 1886 | 1989 | 103 | EN | not specified |
| *Petinomys fuscocapillus* | 1889 | 1989 | 100 | NT | holotype |
| *Phaenomys ferrugineus* | 1940 | 1998 | 58 | VU | time |
| *Phoniscus papuensis* | 1897 | 1981 | 84 | LC | time |
| *Phyllomys unicolor* | 1824 | 2004 | 180 | CR | holotype |
| *Phyllonycteris aphylla* | 1898 | 1957 | 59 | LC | extinct |
| *Plagiodontia aedium* | 1836 | 1948 | 112 | EN | extinct |
| *Porcula salvania* | 1965 | 1971 | 6 | CR | extinct |
| *Potorous gilbertii* | 1900 | 1994 | 94 | CR | extinct |
| *Prionailurus planiceps* | 1985 | 1995 | 10 | EN | time |
| *Prolemur simus* | 1955 | 1986 | 31 | CR | extinct |
| *Pseudantechinus mimulus* | 1906 | 1967 | 61 | EN | holotype |
| *Pseudomys novaehollandiae* | 1887 | 1967 | 80 | VU | extinct |
| *Pteralopex anceps* | 1925 | 1995 | 70 | EN | extinct |
| *Pteropus gilliardorum* | 1959 | 1994 | 35 | DD | holotype |
| *Pteropus nitendiensis* | 1926 | 2001 | 75 | EN | not specified |
| *Rattus giluwensis* | 1983 | 2005 | 22 | DD | time |
| *Rhagomys rufescens* | 1917 | 2003 | 86 | NT | extinct |
| *Rhinolophus maclaudi* | 1967 | 2007 | 40 | EN | time |
| *Rhinolophus osgoodi* | 1939 | 2003 | 64 | DD | holotype |
| *Rhinopithecus avunculus* | 1912 | 1992 | 80 | CR | holotype |
| *Rhynchomys isarogensis* | 1981 | 1988 | 7 | VU | time |
| *Rucervus eldii* | NA | 1975 | NA | EN | extinct |
| *Santamartamys rufodorsalis* | 1899 | 2008 | 109 | DD | holotype |
| *Sminthopsis longicaudata* | 1935 | 1984 | 49 | LC | extinct |
| *Sminthopsis psammophila* | 1894 | 1969 | 75 | EN | extinct |
| *Solenodon cubanus* | 1890 | 1975 | 85 | EN | extinct |
| *Solenodon paradoxus* | NA | 2007 | NA | EN | extinct |
| *Sus bucculentus* | 1892 | 1995 | 103 | DD | holotype |
| *Tarsius pumilus* | 1921 | 2009 | 88 | DD | extinct |
| *Tokudaia muenninki* | 1978 | 2008 | 30 | CR | time |
| *Tolypeutes tricinctus* | 1973 | 1993 | 20 | VU | extinct |
| *Viverra civettina* | 1971 | 1987 | 16 | CR | extinct |
| *Zaglossus attenboroughi* | 1961 | 2007 | 46 | CR | holotype |
| *Zyzomys pedunculatus* | 1966 | 1996 | 30 | CR | extinct |
