# Supplementary material for: The World's Rediscovered Species: Back from the Brink?
Source: PLoS One. 2011 Jul 27;6(7):e22531. doi: 10.1371/journal.pone.0022531 (PMC3144889; doi:10.1371/journal.pone.0022531)
Supplement: Table S2 — Binomial GLMs were used to investigate the relationship between year of rediscovery and years gone missing and whether or not a species was threatened according to IUCN Red List status. Predictor terms shown are year rediscovered and total number of years gone missing. We used a binomial response coded as threatened or non-threatened. Also shown are the number of parameters (k), log likelihood (LL), the difference in AICc of each model from the highest ranked model (ΔAICc), AICc weights representing the probability of each model being the best (wAICc), and the percent deviance explained by each model (%DE). Models are ranked by AICc weights. (DOC) [file pone.0022531.s007.doc]

**Table S2.** The rediscovery year or the number of years missing poorly predicts whether or not a species is threatened.

**Threatened/Non-Threatened ~** Year Rediscovered

|  | **Model** | ***k*** | ***LL*** | **AIC*_c_*** | **∆AIC*_c_*** | ***w*AIC*_c_*** | **%DE** |
| --- | --- | --- | --- | --- | --- | --- | --- |
| *All Species (N = 279)* | |  |  |  |  |  |  |
|  | ~Year Rediscovered | 2 | -198.031 | 202.075 | 0 | 0.799 | 2.363 |
|  | Null | 1 | -202.824 | 204.838 | 2.763 | 0.201 |  |
| *Amphibians (N = 69)* | |  |  |  |  |  |  |
|  | ~Year Rediscovered | 2 | -18.532 | 41.246 | 0 | 0.688 | 9.090 |
|  | Null | 1 | -20.385 | 42.830 | 1.584 | 0.311 |  |
| *Birds (N = 130)* | |  |  |  |  |  |  |
|  | Null | 1 | -48.491 | 99.014 | 0 | 0.732 |  |
|  | ~Year Rediscovered | 2 | -48.469 | 101.034 | 2.019 | 0.267 | 0.045 |
| *Mammals (N = 80)* | |  |  |  |  |  |  |
|  | ~Year Rediscovered | 2 | -58.752 | 62.908 | 0 | 0.832 | 8.292 |
|  | Null | 1 | -64.064 | 66.115 | 3.208 | 0.167 |  |

**Threatened/Non-Threatened ~** Years Gone Missing (i.e., Year Rediscovered – Year Last Seen)

|  | **Model** | ***k*** | ***LL*** | **AIC*_c_*** | **∆AIC*_c_*** | ***w*AIC*_c_*** | **%DE** |
| --- | --- | --- | --- | --- | --- | --- | --- |
| *All Species (N = 269)* | |  |  |  |  |  |  |
|  | ~Years Gone Missing | 2 | -95.213 | 194.473 | 0 | 0.872 | 2.985 |
|  | Null | 1 | -98.144 | 198.302 | 3.830 | 0.128 |  |
| *Amphibians (N = 66)* | |  |  |  |  |  |  |
|  | ~Years Gone Missing | 2 | -18.892 | 41.974 | 0 | 0.537 | 6.039 |
|  | Null | 1 | -20.106 | 42.274 | 0.300 | 0.463 |  |
| *Birds (N = 130)* | |  |  |  |  |  |  |
|  | ~Years Gone Missing | 2 | -47.362 | 98.818 | 0 | 0.524 | 2.329 |
|  | Null | 1 | -48.491 | 99.014 | 0.196 | 0.476 |  |
| *Mammals (N = 73)* | |  |  |  |  |  |  |
|  | Null | 1 | -29.160 | 60.377 | 0.599 | 0.600 |  |
|  | ~Years Gone Missing | 2 | -28.507 | 61.186 | 0.400 | 0.400 | 2.239 |
