# Supplementary material for: The World's Rediscovered Species: Back from the Brink?
Source: PLoS One. 2011 Jul 27;6(7):e22531. doi: 10.1371/journal.pone.0022531 (PMC3144889; doi:10.1371/journal.pone.0022531)
Supplement: Table S3 — The estimate, standard error (SE), z-value, and p-value for each parameter included in each model. The binomial GLMs used threatened or non-threatened as the response variable and year rediscovered and number of years gone missing as predictor variables. (DOC) [file pone.0022531.s008.doc]

**Table S3.** Generalized linear model analysis to evaluate whether the year of rediscovery or total number of years missing determines whether or not a species is threatened.

|  | ***Parameters*** | ***Estimate*** | ***SE*** | **Z-value** | **P** |
| --- | --- | --- | --- | --- | --- |
| ***Year Rediscovered*** | |  |  |  |  |
| *All Species* | Intercept | -36.485 | 16.752 | -2.178 | 0.029 |
|  | Year Rediscovered | 0.019 | 0.008 | 2.295 | 0.022 |
| *Amphibians* | Intercept | -87.596 | 44.127 | -1.985 | 0.047 |
|  | Year Rediscovered | 0.045 | 0.022 | 2.034 | 0.042 |
| *Birds* | Intercept | -3.447 | 25.412 | -0.136 | 0.892 |
|  | Year Rediscovered | 0.002 | 0.012 | 0.213 | 0.831 |
| *Mammals* | Intercept | -81.474 | 36.635 | -2.224 | 0.026 |
|  | Year Rediscovered | 0.042 | 0.018 | 2.270 | 0.023 |
| ***Number of Years Gone Missing*** | |  |  |  |  |
| *All Species* | Intercept | 2.627 | 0.337 | 7.790 | <0.001 |
|  | Years Missing | -0.010 | 0.004 | -2.454 | 0.014 |
| *Amphibians* | Intercept | 3.176 | 0.789 | 4.021 | <0.001 |
|  | Years Missing | -0.015 | 0.010 | -1.583 | 0.113 |
| *Birds* | Intercept | 2.526 | 0.476 | 5.302 | <0.001 |
|  | Years Missing | -0.008 | 0.005 | -1.543 | 0.123 |
| *Mammals* | Intercept | 2.422 | 0.632 | 3.828 | <0.001 |
|  | Years Missing | -0.009 | 0.008 | -1.177 | 0.239 |
