# Supplementary material for: The World's Rediscovered Species: Back from the Brink?
Source: PLoS One. 2011 Jul 27;6(7):e22531. doi: 10.1371/journal.pone.0022531 (PMC3144889; doi:10.1371/journal.pone.0022531)
Supplement: Table S4 — Rediscovered species are highly range-restricted independent of the years gone missing. Generalized linear models were used to investigate the relationship between species range size and the number of years a species went missing (Table A). The models are ranked by Akaike's Information Criterion corrected for small sample size (AICc). Predictor terms shown in Table A are Year = number of years missing and class (i.e., amphibian, bird, or mammal) as a fixed effect. Also shown are the number of parameters (k), log likelihood (LL), the difference in AICc of each model from the highest ranked model (ΔAICc), AICc weights representing the probability of each model being the best (wAICc), and the percent deviance explained by each model (%DE). Table B provides the estimate, standard error (SE), z-value, and p-value for each parameter included in each model. (DOC) [file pone.0022531.s009.doc]

**Table S4 A and B.** Rediscovered species are highly range-restricted independent of the years gone missing.

(A)

| **All rediscovered species (N=309)** | | | | | | | | |
| --- | --- | --- | --- | --- | --- | --- | --- | --- |
| **Rank** | **Model** | ***k*** | ***LL*** | **AIC*_c_*** | **∆AIC*_c_*** | ***w*AIC*_c_*** | **%DE** | |
| 1 | ~Year + class | 4 | -461.861 | 931.853 | 0 | 0.598 | 5.266 | |
| 2 | ~class | 3 | -463.300 | 932.679 | 0.826 | 0.396 | 4.379 | |
| 3 | ~Year | 2 | -469.139 | 942.318 | 10.466 | ≈0 | 0.696 | |
| 4 | ~Null | 1 | -470.218 | 942.450 | 10.597 | ≈0 |  | |
|  | | | | | | | |  |

(B)

| ***Models*** | ***Parameters*** | ***Estimate*** | ***SE*** | **t** | **P** |
| --- | --- | --- | --- | --- | --- |
| ~Year + class | Intercept | 2.682 | 0.140 | 19.176 | <0.001 |
|  | Year Missing | -0.003 | 0.002 | -1.690 | 0.092 |
|  | Birds | 0.510 | 0.150 | 3.399 | <0.001 |
|  | Mammals | 0.057 | 0.161 | 0.357 | 0.721 |
| ~class | Intercept | 2.545 | 0.114 | 22.290 | <0.001 |
|  | Birds | 0.489 | 0.150 | 3.264 | 0.001 |
|  | Mammals | 0.036 | 0.161 | 0.224 | 0.823 |
| ~Year | Intercept | 2.888 | 0.111 | 25.997 | <0.001 |
|  | Years Missing | -0.002 | 0.002 | -1.467 | 0.143 |
